# Efficacy differences between SNM and various peripheral nerve electrical stimulation modalities for NLUTD: a Bayesian network meta-analysis

**DOI:** 10.3389/fneur.2026.1958819

**Published:** 2026-09-21

**Authors:** Fei Miao, Xinhao Wang, Jiong Zhang, Jipeng Wang, Jinfeng Wu, Yong Xia, Jiawen Wang

**Affiliations:** 1 Department of Obstetrics and Gynecology, Fuzhou First General Hospital Affiliated with Fujian Medical University, Fuzhou, China; 2 Department of Urology, Beijing Hospital, National Center of Gerontology, Institute of Geriatric Medicine, Beijing, China; 3 Department of Urology, Fuzhou University Affiliated Provincial Hospital, Fujian Provincial Hospital, Fuzhou, China; 4 Pelvic Floor Dysfunction Diagnosis and Treatment Center, Fuzhou First General Hospital affiliated with Fujian Medical University, Fuzhou, China

**Keywords:** Bayesian model, network meta-analysis, neuromuscular electrical stimulation, SNM, tibial nerve stimulation, transcutaneous electrical nerve stimulation

## Abstract

**Background:**

Neurogenic lower urinary tract dysfunction (NLUTD) severely affects patients’ quality of life, and conventional therapies have limited efficacy. Direct comparisons between SNM and peripheral nerve electrical stimulation are lacking. This study employed a network meta-analysis to evaluate the efficacy of these two approaches on voiding-related parameters.

**Methods:**

A computerized search of PubMed, Embase, Scopus, Web of Science, VIP, Wanfang, and CNKI databases was conducted for publicly available randomized controlled trials (RCTs) on sacral neuromodulation and peripheral nerve electrical stimulation for neurogenic lower urinary tract dysfunction, up to May 2026. The Cochrane Handbook version 5.4 (RoB 2) was used for quality assessment of the included studies. Bayesian network meta-analysis was performed using Rstudio (version 4.4.1) to compare and rank the outcomes.

**Results:**

The network meta-analysis showed that for maximum flow rate (Qmax), neuromuscular electrical stimulation (NMES) combined with conventional therapy (SUCRA = 90.5%) ranked highest, significantly better than placebo (MD = 6.5 mL/s, 95% CrI: 0.38, 13) and conventional therapy (MD = 5.6 mL/s, 95% CrI: 3.1, 8.3). For improvement in maximum cystometric capacity (MCC), sacral neuromodulation (SNM) combined with conventional therapy (SUCRA = 96.2%) demonstrated the best efficacy; however, because only six SNM-related RCTs were included, this result should be considered exploratory and cannot yet confirm its superiority over other interventions. In terms of post-void residual (PVR) volume, reduction in 24-h voiding frequency, and improvement in 24-h incontinence episodes, NMES combined with conventional therapy ranked first (SUCRA = 90, 89.5, and 91%, respectively). For mean voided volume (MVV), tibial nerve stimulation (TNS) combined with conventional therapy (SUCRA = 88.6%) performed best. Overall, combination treatment strategies were generally superior to monotherapies, with different techniques showing differential advantages in improving various dimensions of lower urinary tract function.

**Conclusion:**

For improving maximum flow rate, post-void residual volume, 24-h voiding frequency, and incontinence episodes, NMES combined with conventional therapy ranked highest; SNM combined with conventional therapy ranked highest for maximum cystometric capacity; TNS combined with conventional therapy ranked highest for mean voided volume. However, these rankings only suggest potential relative advantages and do not establish definitive superiority; in particular, the evidence for SNM is limited, and the results should be considered exploratory.

**Systematic review registration:**

https://www.crd.york.ac.uk/PROSPERO/, identifier CRD420261324281.

## 1 Introduction

Neurogenic lower urinary tract dysfunction (NLUTD) is a general term for bladder and/or urethral dysfunction caused by neurological disorders or injuries, resulting in impaired voiding and urinary continence function (1). Studies have shown that the prevalence of NLUTD has been increasing in recent years, with prevalence rates of 20–50% in stroke patients and approximately 70–95% in patients with spinal cord injury (2). The clinical manifestations of NLUTD are complex and varied, presenting not only storage symptoms such as urinary urgency, frequency, and urge incontinence, but also voiding symptoms such as dysuria and urinary retention (3). It not only severely affects patients’ quality of life but also imposes a heavy socioeconomic burden on the healthcare system.

Traditional treatment strategies for NLUTD include behavioral interventions, pharmacotherapy, intermittent catheterization, and surgical procedures. However, pharmacotherapy has limited efficacy or tolerability issues in some patients, whereas more invasive surgical interventions require careful weighing of risks and benefits. Given these limitations, neuromodulation techniques have emerged as a promising minimally invasive treatment option and have gained increasing attention in the management of NLUTD.

Sacral neuromodulation (SNM), an emerging minimally invasive treatment technique, delivers continuous low-intensity electrical stimulation to the sacral nerves via an implanted stimulator to modulate bladder and urethral neural reflexes, thereby restoring bladder storage and voiding function (4). This technique has been increasingly recognized and applied in clinical practice owing to its minimally invasive nature and individualized treatment characteristics (5). Peripheral nerve electrical stimulation techniques have also been extensively studied in the treatment of NLUTD because of their advantages of being noninvasive or minimally invasive, reversible, and relatively low in cost. These techniques include neuromuscular electrical stimulation (NMES), tibial nerve stimulation (TNS), and transcutaneous electrical nerve stimulation (TENS), among others. They are believed to modulate bladder function by stimulating somatic afferent fibers, thereby inhibiting detrusor overactivity via central reflex pathways or strengthening pelvic floor muscles through efferent stimulation (6). After reviewing the relevant literature, we identified the four most commonly used neuromodulation techniques in the clinical treatment of NLUTD: sacral neuromodulation, neuromuscular electrical stimulation, tibial nerve stimulation, and transcutaneous electrical nerve stimulation. Currently, there is a lack of data on efficacy differences between sacral neuromodulation and various peripheral nerve electrical stimulation techniques, making it difficult to address complex treatment scenarios. Network meta-analysis constructs multi-arm comparison models to enable direct and indirect comparisons of efficacy differences among different neuromodulation techniques across studies, thereby providing evidence-based guidance for clinicians to select the optimal neuromodulation technique when direct comparative evidence is lacking.

## 2 Materials and methods

### 2.1 Databases and literature search strategy

We conducted a computerized search of PubMed, Embase, Scopus, Web of Science, VIP, Wanfang, and CNKI databases for randomized controlled trials (RCTs) related to this study, with a search timeframe up to May 2026. The main search terms included SNM, neuromuscular electrical stimulation (NMES), tibial nerve stimulation (TNS), transcutaneous electrical nerve stimulation (TENS), and other keywords. Supplementary Table 1 describes the comprehensive search strategy.

### 2.2 Inclusion criteria

- Study type: Randomized controlled trials (RCTs) published before May 2026 comparing different nerve electrical stimulation therapies, or comparing nerve electrical stimulation therapy with control treatment for neurogenic lower urinary tract dysfunction, regardless of whether blinding was used. The design, implementation, and reporting of this study followed the Preferred Reporting Items for Systematic Reviews and Network Meta-Analyses (PRISMA-NMA) guidelines.
- Participants: Patients with a confirmed diagnosis of neurogenic lower urinary tract dysfunction, meeting either (1) the 2002 International Continence Society (ICS) report on the standardization of terminology of lower urinary tract function: report from the Standardization Sub-Committee of the International Continence Society (7); or (2) the classification criteria published by the Urology Branch of the Chinese Medical Association (CUA) (8); with no restrictions on age or sex.
- Interventions: Comparisons between different nerve electrical stimulation modalities combined with conventional therapy (intermittent catheterization plus pelvic floor muscle training or pharmacotherapy); comparisons between nerve electrical stimulation combined with conventional therapy and conventional therapy alone; and comparisons between different nerve electrical stimulation modalities and placebo or conventional therapy.
- Outcome measures: ① maximum flow rate (Qmax, mL/s); ② maximum cystometric capacity (MCC, mL); ③ post-void residual volume (PVR, mL); ④ mean voided volume (MVV, mL); ⑤ 24-h voiding frequency (voids/24 h); ⑥ 24-h incontinence episodes (episodes/24 h).

### 2.3 Exclusion criteria

- Non-Chinese or non-English literature.
- Participants without a confirmed diagnosis of neurogenic lower urinary tract dysfunction meeting the inclusion criteria.
- Missing, erroneous, or unobtainable key outcome data despite contacting the authors; duplicate publications.
- Case reports, case series, animal studies, or reviews.

### 2.4 Literature screening and data extraction

EndNote software was used for duplicate removal and initial screening of retrieved records; full texts were then downloaded and screened to exclude studies not meeting the inclusion criteria. If key data were missing, the original authors could be contacted. The above procedures were performed independently by two investigators using pre-designed forms, and data were extracted; any disagreements were resolved through consultation with a third investigator. Non-English literature was independently translated by bilingual researchers with medical backgrounds, and the accuracy of key terminology was reviewed by relevant experts. An Excel spreadsheet was established for data extraction, including first author, publication year, sample size (male/female), age, interventions, NMES application mode, treatment duration, and outcome measures.

### 2.5 Risk of bias assessment

We used the Cochrane risk-of-bias tool (RoB 2) to assess the risk of bias in the included studies. The assessment domains included random sequence generation, allocation concealment, blinding of participants and investigators, blinding of outcome assessors, incomplete outcome data, selective reporting, and other biases. The GRADE system was used to rate the strength of the body of evidence. In the evidence quality assessment, five major downgrading factors were considered (risk of bias, imprecision, inconsistency, indirectness, and publication bias), and the overall quality of evidence was classified as high, moderate, low, or very low.

### 2.6 Statistical analysis

Based on a Bayesian framework, R software (e.g., the gemtc package) was used to construct network meta-analysis models. Continuous outcomes were expressed as mean differences (MD) with 95% credible intervals (95% CrI); dichotomous variables were expressed as odds ratios (OR) or risk ratios (RR) with 95% CrI. The I^2^ statistic was used to assess heterogeneity among studies; if I^2^ ≥ 70%, substantial heterogeneity was considered present, and subgroup analyses and sensitivity analyses were performed to explore the sources of heterogeneity. If the source could not be identified, a random-effects model was used based on methodological and statistical considerations. For model construction, four Markov chains were set with different initial values; the burn-in period was set to 5,000 iterations to eliminate the influence of initial values, followed by 20,000 effective iterations. The gelman.diag() function in R was used to calculate the R-hat value (potential scale reduction factor, PSRF) to assess model convergence; an R-hat close to 1.0 indicated adequate convergence, otherwise iterations were increased to 50,000 and convergence was reassessed. The deviance information criterion (DIC) was used to select between random-effects and fixed-effects models; DIC values were calculated for both, and the model with the smaller DIC was generally selected as the optimal model (9). If the network contained closed loops, the node-splitting method was used to assess inconsistency between direct and indirect evidence (10). Small-study effects were assessed using comparison-adjusted funnel plots. Finally, interventions were ranked using the surface under the cumulative ranking curve (SUCRA); higher SUCRA percentages generally indicate better efficacy (11).

## 3 Results

### 3.1 Literature search results

Initial search retrieved 9,404 records; 8,827 duplicate records were removed; 489 records were excluded after screening titles and abstracts; 45 records were excluded after full-text review for not meeting the inclusion criteria; and 11 records with controversial issues were removed. Finally, 32 studies (12–43) were included, of which 13 were in English (15, 16, 25–33, 35, 36) and 19 were in Chinese (12–14, 17–24, 34, 37–43). The literature screening process is shown in Figure 1.

**Figure 1:**
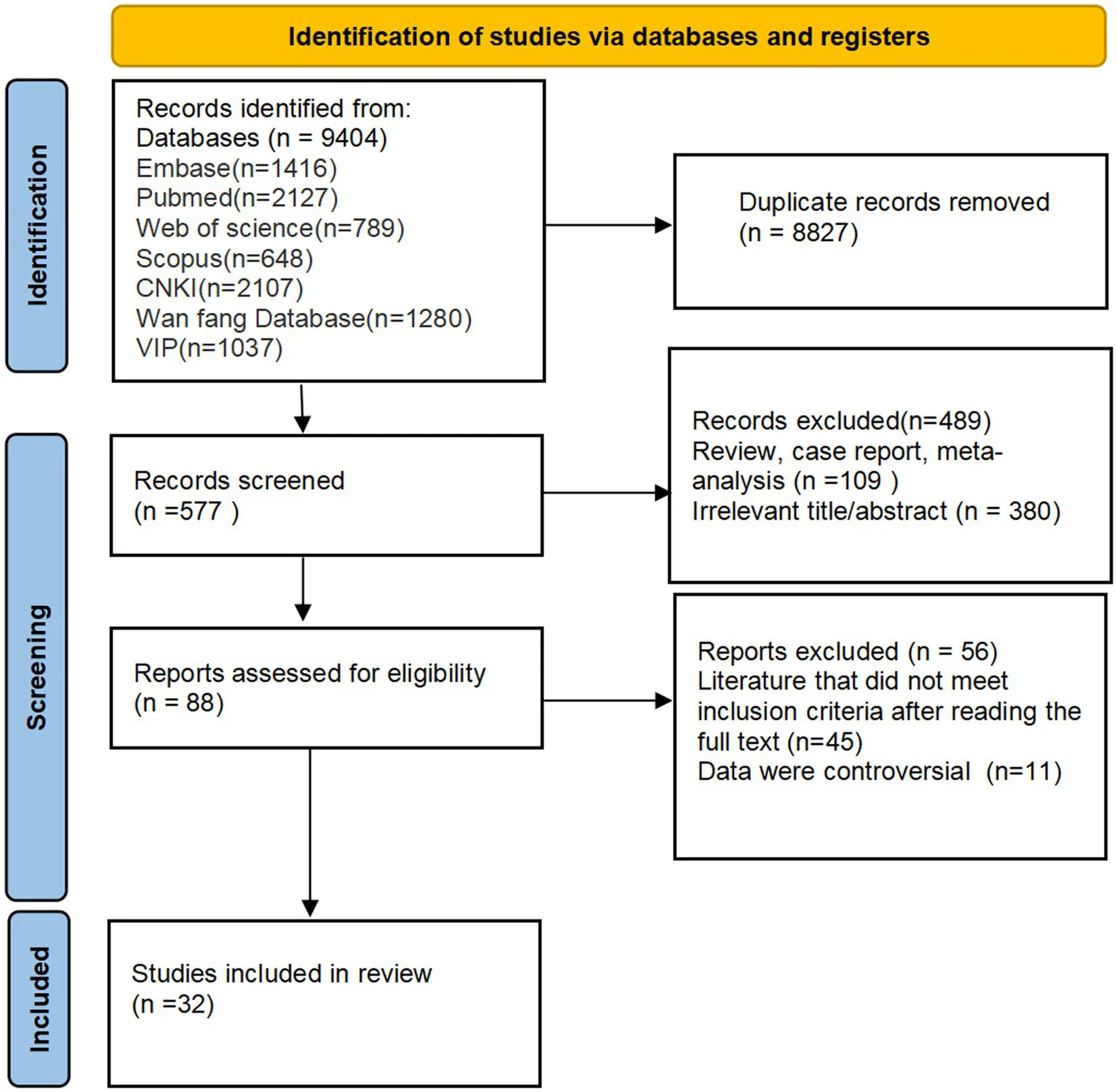
Flow chart of PRISMA.

### 3.2 Characteristics of included studies and risk of bias assessment

A total of 32 studies (12–43) with 2,266 participants were included; 31 were two-arm studies and 1 was a three-arm study. Regarding risk of bias, 29 studies described specific randomization methods (12–16, 18–34, 36–43), 8 studies used opaque sealed envelopes (15, 16, 19, 25, 30, 31, 33, 36) and 12 studies used double- or single-blinding (15, 16, 25–33, 36). The risk of bias due to incomplete outcome data was low, while other risks of bias and selective reporting were rated as unclear. The basic characteristics of the included studies are shown in Table 1. Because the implementation route of NMES may vary, we summarized the intervention implementation characteristics of NMES in each included study in Supplementary Table 2, including the route of administration, electrode placement, and target muscle groups. Risk of bias assessment is shown in Figure 2 and Supplementary Figure 1. We also performed GRADE assessments for the six primary outcomes across all intervention comparisons; detailed results are presented in Supplementary Table 3. Overall, the quality of evidence was moderate for most comparisons. The main reasons for downgrading included: (1) risk of bias—most studies had a high or unclear risk in blinding and random allocation; (2) imprecision—in some comparisons, the 95% credible intervals were wide and crossed the null effect line, indicating imprecise estimates.

**Table 1:** Characteristics of the included studies.

Author (year)	N (M/F)	Average age/year	Intervention		Stimulation frequency (Hz)	Pulse width (μs)	Stimulation amplitude/pulse current	Stimulation duration	Treatment course	Outcome indicators
	T/C	T/C	T/C							
XX Liu 2020 (12)	(24/16) (28/12)	52.82 ± 7.75 51.54 ± 7.58	SNM + CIC + PFMT	CIC + PFMT	14	210	As tolerated by the patient	24 h	NA	①②③④⑤⑥
XJ Bai 2025 (13)	(35/21) (38/18)	46.97 ± 12.26 47.64 ± 10.90	SNM + CIC + PFMT	CIC + PFMT	14	210	As tolerated by the patient	24 h	NA	①②③④⑤⑥
L You 2023 (14)	(32/17) (36/13)	10.75 ± 1.12 11.03 ± 1.28	SNM + CIC + PFMT	CIC + PFMT	14	210	As tolerated by the patient	24 h	6 months	①②④⑤
Liechti MD 2022 (15)	(7/22) (10/21)	50 49	SNM	PLA	14	210	As tolerated by the patient	24 h	4 months	①②③④⑤⑥
Guys JM 2004 (16)	(26/16)	11.9 11.9	SNM	CIC + PFMT	14	210	As tolerated by the patient	24 h	6 months	①②③④⑤⑥
TL Fan 2016 (17)	(17/47)	50.5 ± 14.8	SNM + CIC + PFMT	CIC + PFMT	14	210	As tolerated by the patient	24 h	NA	①②③④⑤⑥
DM Xu 2015 (18)	(19/6) (18/7)	43.52 ± 10.86 39.80 ± 11.65	NMES+CIC + PFMT	CIC + PFMT	20	200	As tolerated by the patient	30 min	8 weeks	②③④
LT Chen 2016 (19)	(16/4) (15/5)	41 ± 12 40 ± 11	NMES+CIC + PFMT	CIC + PFMT	20	NA	As tolerated by the patient	30 min	8 weeks	③④⑤
SX Chen 2014 (20)	(21/15) (19/17)	45.1 ± 2.6 44.4 ± 1.5	NMES+CIC + PFMT	CIC + PFMT	20	200	As tolerated by the patient	30 min	6 weeks	③④⑤
HQ Zhou 2020 (21)	(24/18) (26/16)	39.53 ± 4.45 38.92 ± 5.03	NMES+CIC + PFMT	CIC + PFMT	NA	NA	As tolerated by the patient	20 min	8 weeks	①③④
SG Pan 2016 (22)	(28/15) (30/13)	38.6 ± 13.2 40.2 ± 15.2	NMES+CIC + PFMT	CIC + PFMT	20	200	As tolerated by the patient	30 min	8 weeks	①②③
XL An 2014 (23)	(34/29) (35/28)	64.0 ± 4.0 63.4 ± 3.9	NMES+CIC + PFMT	CIC + PFMT	20	NA	As tolerated by the patient	40 min	4 weeks	②③④⑤
ZL Ma 2021 (24)	(29/20) (25/19)	35.18 ± 10.63 34.57 ± 9.83	NMES+CIC + PFMT	CIC + PFMT	20	NA	As tolerated by the patient	20 min	6 weeks	③④⑤⑥
McClurg D 2008 (25)	(6/31) (11/26)	48.3 ± 11.5 52.0 ± 8.8	NMES+CIC + PFMT	PLA + CIC + PFMT	10	450	As tolerated by the patient	30 min	9 weeks	①③④⑥
Lúcio A 2016^a^ (26)	(0/10)(0/10)(0/10)	42/45/43.5	NMES+CIC + PFMT/TNS + CIC + PFMT/PLA + CIC + PFMT		10	200	As tolerated by the patient	30 min	12 weeks	①②③④⑤⑥
Castel-Lacanal E,2025 (27)	(32/15) (39/14)	68 ± 8.7 69.1 ± 8	TNS	PLA	10	200	As tolerated by the patient	20 min	12 weeks	①②③⑥
Stampas A 2019 (28)	(8/4) (2/5)	48.81 ± 9.65 48.97 ± 10.84	TNS	PLA	10	200	As tolerated by the patient	30 min	1.4 months	①②③④⑤⑥
Marzouk MH 2022 (29)	(10/10) (11/9)	69.5 ± 7.7 68.9 ± 8.2	TNS	PLA	10	200	As tolerated by the patient	30 min	8 weeks	①②③④⑤⑥
Monteiro ÉS 2014 (30)	(12/0) (12/0)	56.1 ± 10.5 65.4 ± 9.8	TNS	CIC + PFMT	10	200	As tolerated by the patient	30 min	6 weeks	④⑤⑥
Perissinotto MC 2015 (31)	NA NA	63.5 57.0	TNS	PLA	10	200	As tolerated by the patient	30 min	5 weeks	①②③④⑤⑥
Araujo TG 2021 (32)	(0/15) (0/15)	64.2 ± 2.5 68.2 ± 2.3	TNS	PLA	10	200	As tolerated by the patient	20 min	12 weeks	⑤⑥
Liu Y 2022 (33)	(21/20) (22/21)	63.6 ± 4.3 62.9 ± 3.6	TENS	anticholinergic drugs + CIC + PFMT	75	150	As tolerated by the patient	30 min	8 weeks	①②③④⑤⑥
JL Wang 2024 (34)	(21/33) (19/35)	36.02 ± 2.95 35.36 ± 3.22	TENS + CIC + PFMT	CIC + PFMT	20	NA	NA	20 min	4 weeks	①②③④⑤⑥
Guo ZF 2014 (35)	(21/11) (21/8)	68.1 ± 7.1 65.1 ± 9.8	TENS + CIC + PFMT	CIC + PFMT	75	70	As tolerated by the patient	30 min	8 weeks	①②⑤⑥
Liu Y 2016 (36)	(19/8) (22/5)	62.6 ± 3.3 61.9 ± 2.6	TENS	PLA	75	150	As tolerated by the patient	30 min	12 weeks	①②③④⑤⑥
XY Li 2018 (37)	(39/14) (40/13)	41.21 ± 9.17 40.32 ± 10.13	TENS	CIC + PFMT	20	300	NA	30 min	4 weeks	①②③④⑤⑥
MH Yu 2023 (38)	(15/6) (13/8)	41.3 ± 19.6 37.4 ± 17.9	TENS + CIC + PFMT	PLA + CIC + PFMT	20	NA	As tolerated by the patient	40 min	8 weeks	③④⑤
Y Liu 2022 (39)	(35/7) (31/10)	73. 020 ± 8.312 70. 690 ± 6. 251	TENS	CIC + PFMT	20	150	NA	30 min	8 weeks	①②④⑤⑥
LJ Wang 2024 (40)	(25/19) (23/21)	55.32 ± 4.13 55.43 ± 4.07	TENS + CIC + PFMT	CIC + PFMT	50	NA	NA	20 min	8 weeks	①②③④⑤
YQ Ye 2019 (41)	(25/19) (23/21)	55.32 ± 4.13 55.43 ± 4.07	TENS + CIC + PFMT	CIC + PFMT	50	NA	As tolerated by the patientt	20 min	8 weeks	①②③④⑤
Y Wang 2022 (42)	(29/19) (28/20)	48.22 ± 7.61 48.17 ± 7.92	TENS+CIC + PFMT	CIC + PFMT	50	NA	As tolerated by the patient	20 min	8 weeks	①②③④⑤
GX Chen 2019 (43)	(36/12) (33/15)	34.19 ± 13.46 32.59 ± 12.18	TENS+CIC + PFMT	CIC + PFMT	50	NA	As tolerated by the patient	20 min	4 weeks	②③④⑤

N, sample size; M, male; F, female; T, treatment group; C, control group; ① Maximum flow rate (Qmax); ② Maximum cystometric capacity (MCC); ③ Post-void residual volume (PVR); ④ Mean voided volume (MVV); ⑤ 24-h voiding frequency; ⑥ 24-h incontinence episodes. SNM, Sacral neuromodulation; NMES, Neuromuscular electrical stimulation; TNS, Tibial nerve stimulation; TENS, Transcutaneous electrical nerve stimulation; PLA, Placebo; NA, Not applicable; A, Three-arm study; CIC + PFMT, Functional exercise and intermittent catheterization.

**Figure 2:**
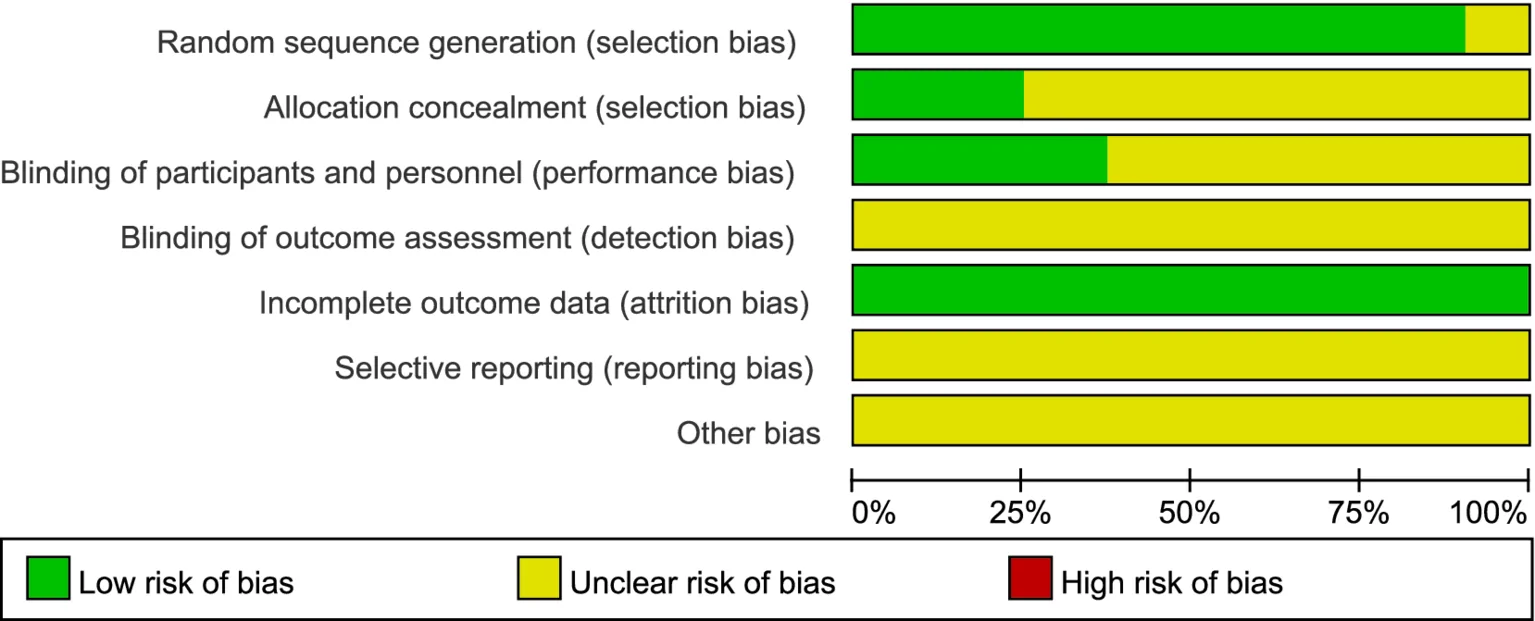
Risk of bias assessment proportion plot.

### 3.3 Convergence assessment and evidence network

We used trace plots, density plots, and Brooks-Gelman-Rubin diagnostic plots to assess convergence. Specifically, the maximum number of iterations in the trace plots was 50,000; most trajectories were stable, indicating that the Markov chains converged well after multiple iterations, with stable estimates and relatively high reliability of the simulation results. The density plots were mostly unimodal; a unimodal distribution indicates a central tendency of values, with the peak corresponding to the most likely value. After 10,000 iterations, the red dashed line representing the 97.5% quantile rapidly approached 1 and stabilized, and the median curve also stabilized near 1. This indicates that all MCMC chains achieved convergence, and the parameter estimates based on these chains were reliable, as shown in Figure 3 and Supplementary Figure 2. Figure 4 shows the network plots for each outcome. As can be seen, closed loops were formed for all outcomes; therefore, we performed inconsistency testing.

**Figure 3:**
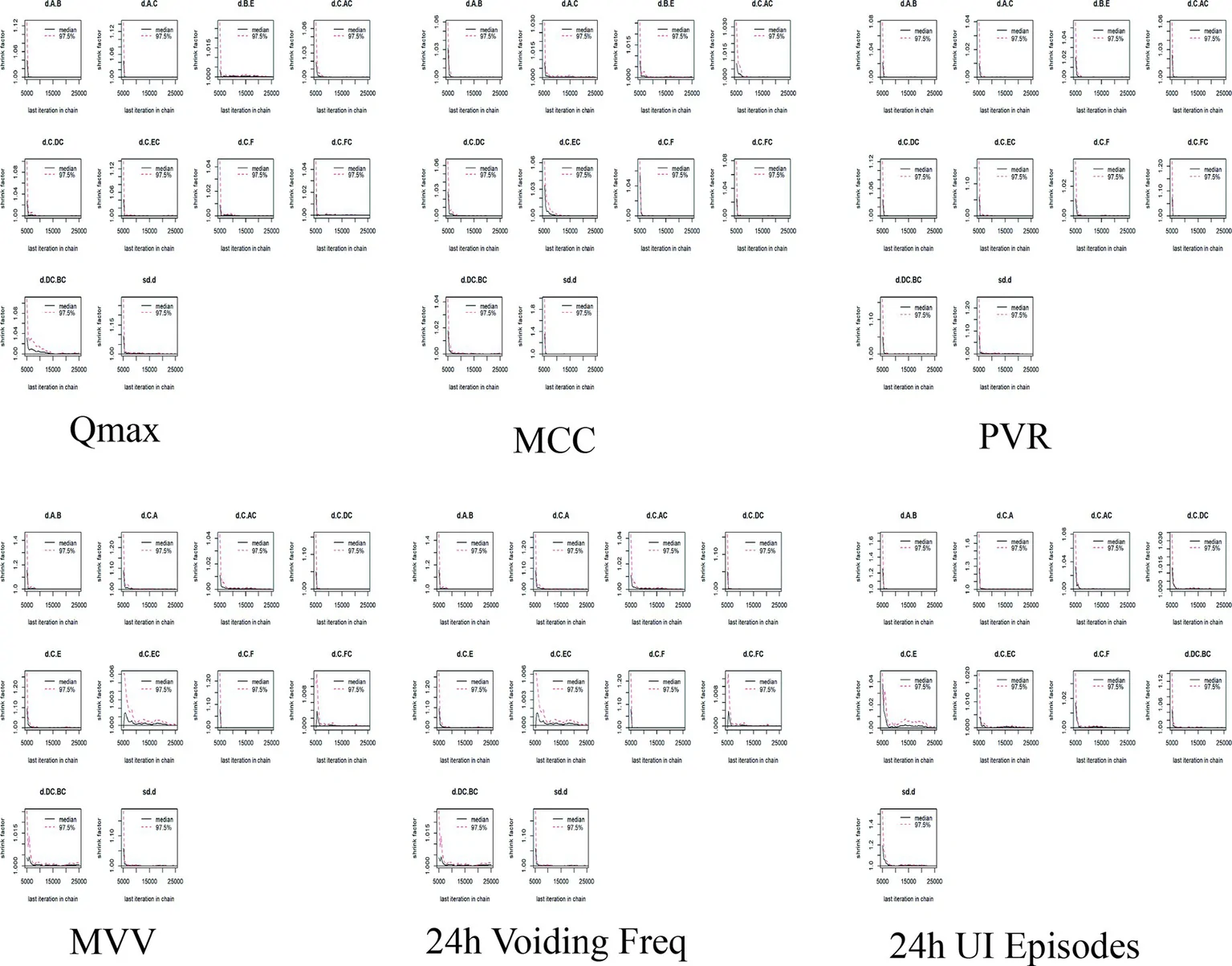
Brooks–Gelman–Rubin diagnostic plot. The horizontal axis represents the number of iterations in the chain, and the vertical axis represents the shrinkage factor. The solid black line represents the median of the shrinkage factor, and the red dashed line represents the 97.5% quantile of the shrinkage factor. When both the median and the 97.5% quantile of the shrinkage factor approach 1 and stabilize after iterations, it indicates good convergence. Qmax, Maximum urinary flow rate; MCC, Maximum cystometric capacity; PVR, Post-void residual volume; MVV, Mean voided volume; 24 h Voiding Freq, 24-h voiding frequency; 24 h UI Episodes, 24-h urinary incontinence episodes; A, Sacral neuromodulation; AC, Sacral neuromodulation combined with conventional therapy; B, Placebo; BC, Placebo combined with conventional therapy; C, Conventional therapy; DC, Neuromuscular electrical stimulation combined with conventional therapy; E, Tibial nerve stimulation; EC, Tibial nerve stimulation combined with conventional therapy; F, Transcutaneous electrical nerve stimulation; FC, Transcutaneous electrical nerve stimulation combined with conventional therapy.

**Figure 4:**
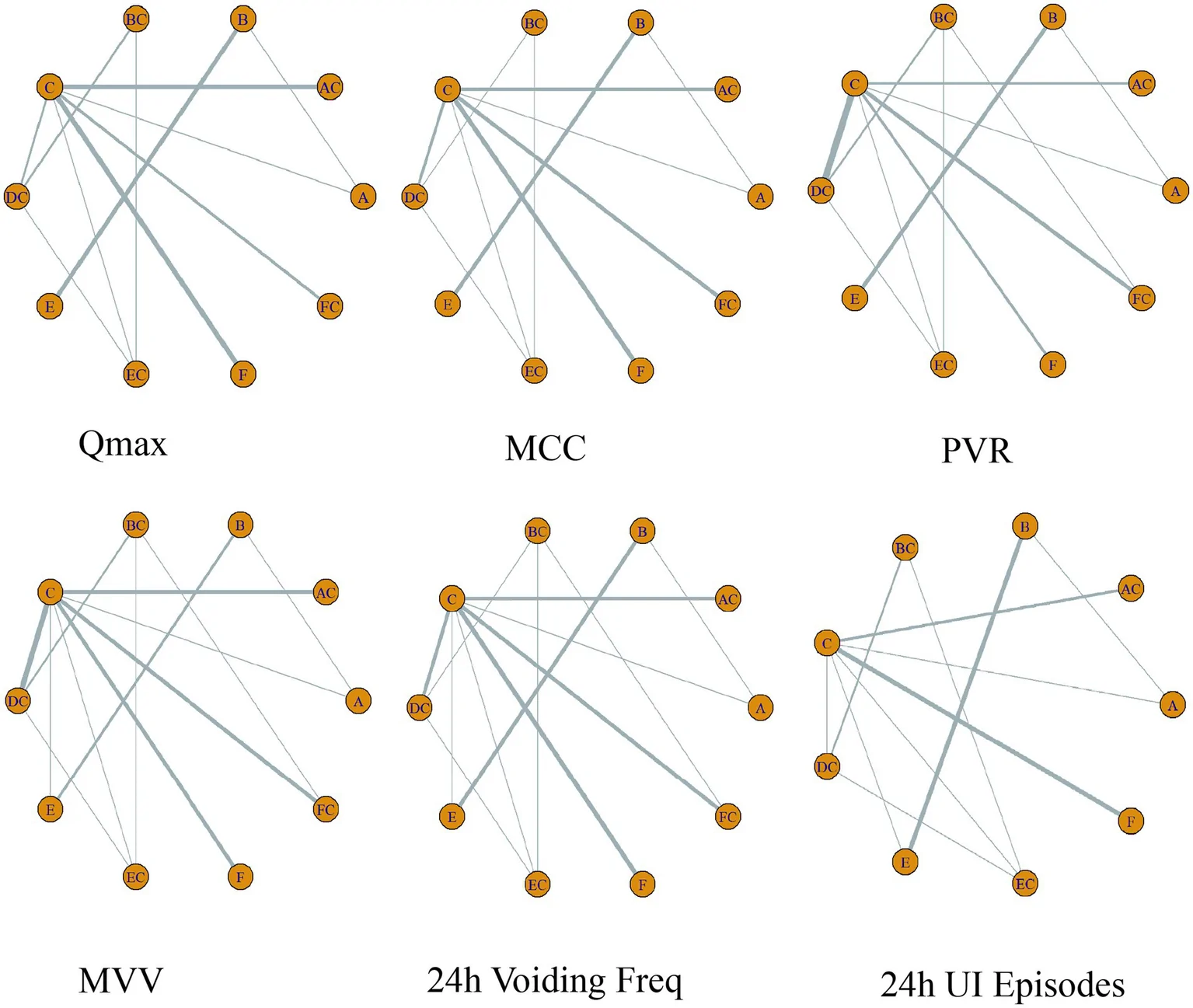
Evidence network for each outcome indicator. Circles represent individual interventions, with the size of the circle corresponding to the sample size. The thickness of the lines represents the number of studies comparing the two connected interventions. A connection between two points indicates a direct comparison between the two interventions. Qmax, Maximum urinary flow rate; MCC, Maximum cystometric capacity; PVR, Post-void residual volume; MVV, Mean voided volume; 24-h Voiding Freq, 24-h voiding frequency; 24 h UI Episodes, 24-h urinary incontinence episodes; A, Sacral neuromodulation; AC, Sacral neuromodulation combined with conventional therapy; B, Placebo; BC, Placebo combined with conventional therapy; C, Conventional therapy; DC, Neuromuscular electrical stimulation combined with conventional therapy; E, Tibial nerve stimulation; EC, Tibial nerve stimulation combined with conventional therapy; F, Transcutaneous electrical nerve stimulation; FC, Transcutaneous electrical nerve stimulation combined with conventional therapy.

### 3.4 Model selection for each outcome

Supplementary Table 4 summarizes the consistency model fit for each outcome under the fixed-effects and random-effects models, including the deviance information criterion (DIC), pD, Dbar, and I^2^. Based on model fit and heterogeneity indices, the optimal model was determined for each outcome for subsequent analyses.

### 3.5 Consistency testing

The node-splitting method was used to examine the consistency of closed-loop comparisons in the network plot; Figure 5 shows the inconsistency test plot. The direction of effect estimates for direct and indirect comparisons was generally consistent across all comparisons, and *p* > 0.05 indicated no significant inconsistency, suggesting that the comparison results were reliable and could be analyzed using a consistency model.

**Figure 5:**
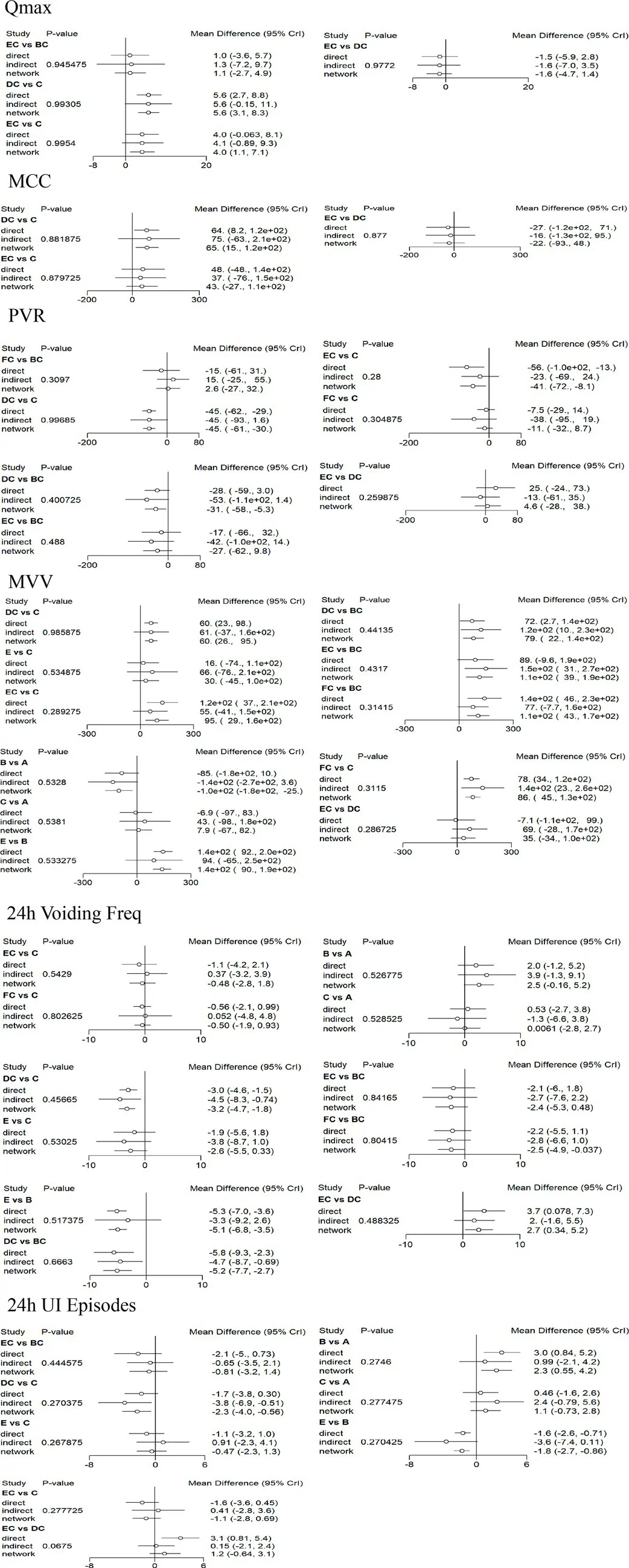
Inconsistency test plot for each outcome indicator. For each comparison, the three points represent the effect estimates from direct comparison, indirect comparison, and network meta-analysis, respectively. The lines extending from the points represent the 95% credible intervals (CrI). Qmax, Maximum urinary flow rate; MCC, Maximum cystometric capacity; PVR, Post-void residual volume; MVV, Mean voided volume; 24 h Voiding Freq, 24-h voiding frequency; 24 h UI Episodes, 24-h urinary incontinence episodes; A, Sacral neuromodulation; AC, Sacral neuromodulation combined with conventional therapy; B, Placebo; BC, Placebo combined with conventional therapy; C, Conventional therapy; DC, Neuromuscular electrical stimulation combined with conventional therapy; E, Tibial nerve stimulation; EC, Tibial nerve stimulation combined with conventional therapy; F, Transcutaneous electrical nerve stimulation; FC, Transcutaneous electrical nerve stimulation combined with conventional therapy.

### 3.6 Network meta-analysis results

#### 3.6.1 Qmax analysis results

Based on the stable evidence network described above, Bayesian network meta-analysis showed that 23 studies reported maximum flow rate (Qmax) (12–17, 21, 22, 25–29, 31, 33–37, 39–42). For Qmax, the analysis results showed that compared with placebo, NMES combined with conventional therapy (MD = 6.5 mL/s; 95% CrI = 0.38, 13) and TNS (MD = 3.4 mL/s; 95% CrI = 1.4, 5.4) both performed significantly better. NMES combined with conventional therapy was superior to SNM (MD = 5 mL/s; 95% CrI = 0.23, 9.9). Compared with conventional therapy, SNM combined with conventional therapy (MD = 3.3 mL/s; 95% CrI = 1.3, 5.3), NMES combined with conventional therapy (MD = 5.6 mL/s; 95% CrI = 3.1, 8.3), TNS combined with conventional therapy (MD = 4.0 mL/s; 95% CrI = 1.0, 7.1), TENS (MD = 3.6 mL/s; 95% CrI = 1.8, 5.3), and TENS combined with conventional therapy (MD = 4.6 mL/s; 95% CrI = 2.3, 6.9) all performed significantly better. No statistically significant differences were observed with other interventions (see Figure 6). According to the surface under the cumulative ranking curve (SUCRA) results: NMES combined with conventional therapy had the highest probability (90.5%), followed by TENS combined with conventional therapy (77.2%), TNS combined with conventional therapy (67%), TENS (60.1%), SNM combined with conventional therapy (54.7%), TNS (50.8%), placebo combined with conventional therapy (50.3%), SNM (24.1%), conventional therapy (14.1%), and placebo (10.6%) (see Figure 7 and Table 2).

**Figure 6:**
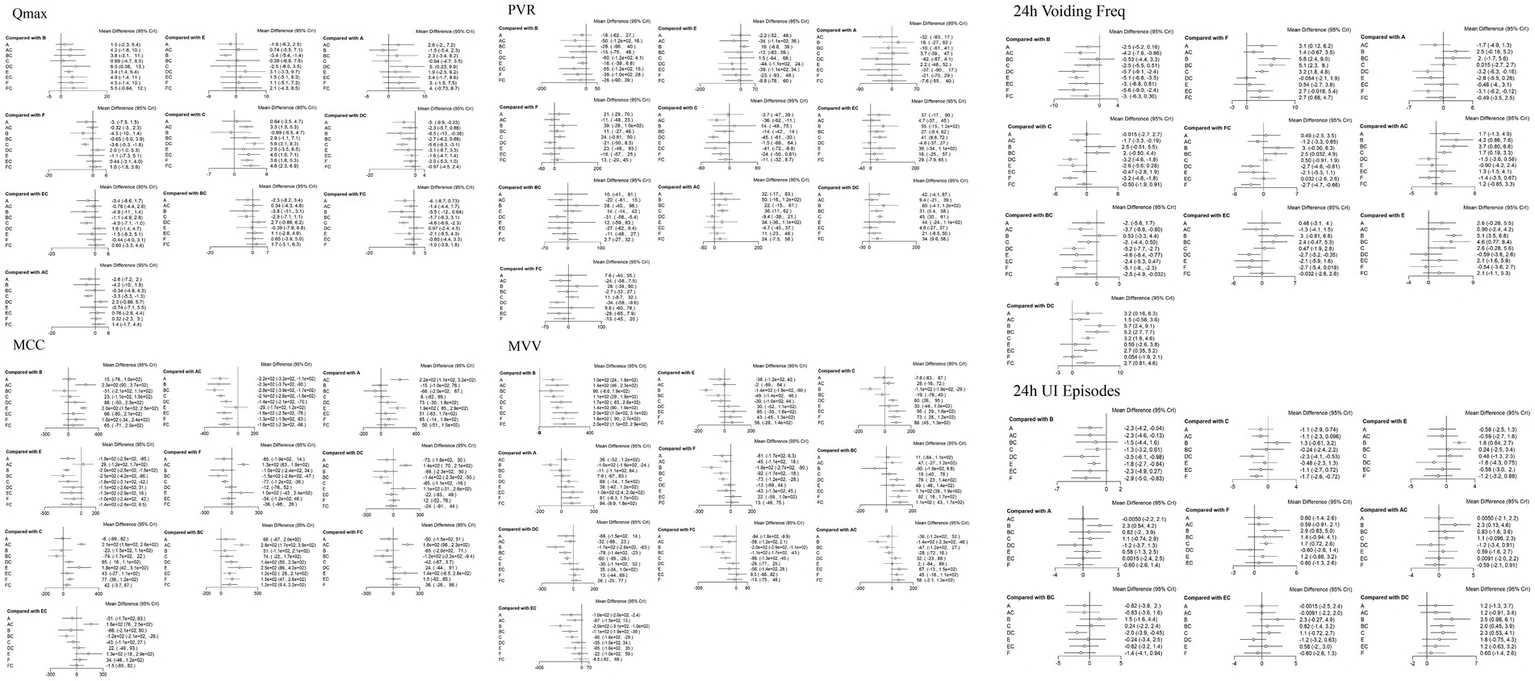
Forest plot for each outcome indicator. Each point represents the odds ratio (*OR*) or mean difference (*MD*) for the intervention, and the lines extending from the points represent the 95% credible intervals (*CrI*). Qmax, Maximum urinary flow rate; MCC, Maximum cystometric capacity; PVR, Post-void residual volume; MVV, Mean voided volume; 24-h Voiding Freq, 24-h voiding frequency; 24-h UI Episodes, 24-h urinary incontinence episodes; A, Sacral neuromodulation; AC, Sacral neuromodulation combined with conventional therapy; B, Placebo; BC, Placebo combined with conventional therapy; C, Conventional therapy; DC, Neuromuscular electrical stimulation combined with conventional therapy; E, Tibial nerve stimulation; EC, Tibial nerve stimulation combined with conventional therapy; F, Transcutaneous electrical nerve stimulation; FC, Transcutaneous electrical nerve stimulation combined with conventional therapy.

**Figure 7:**
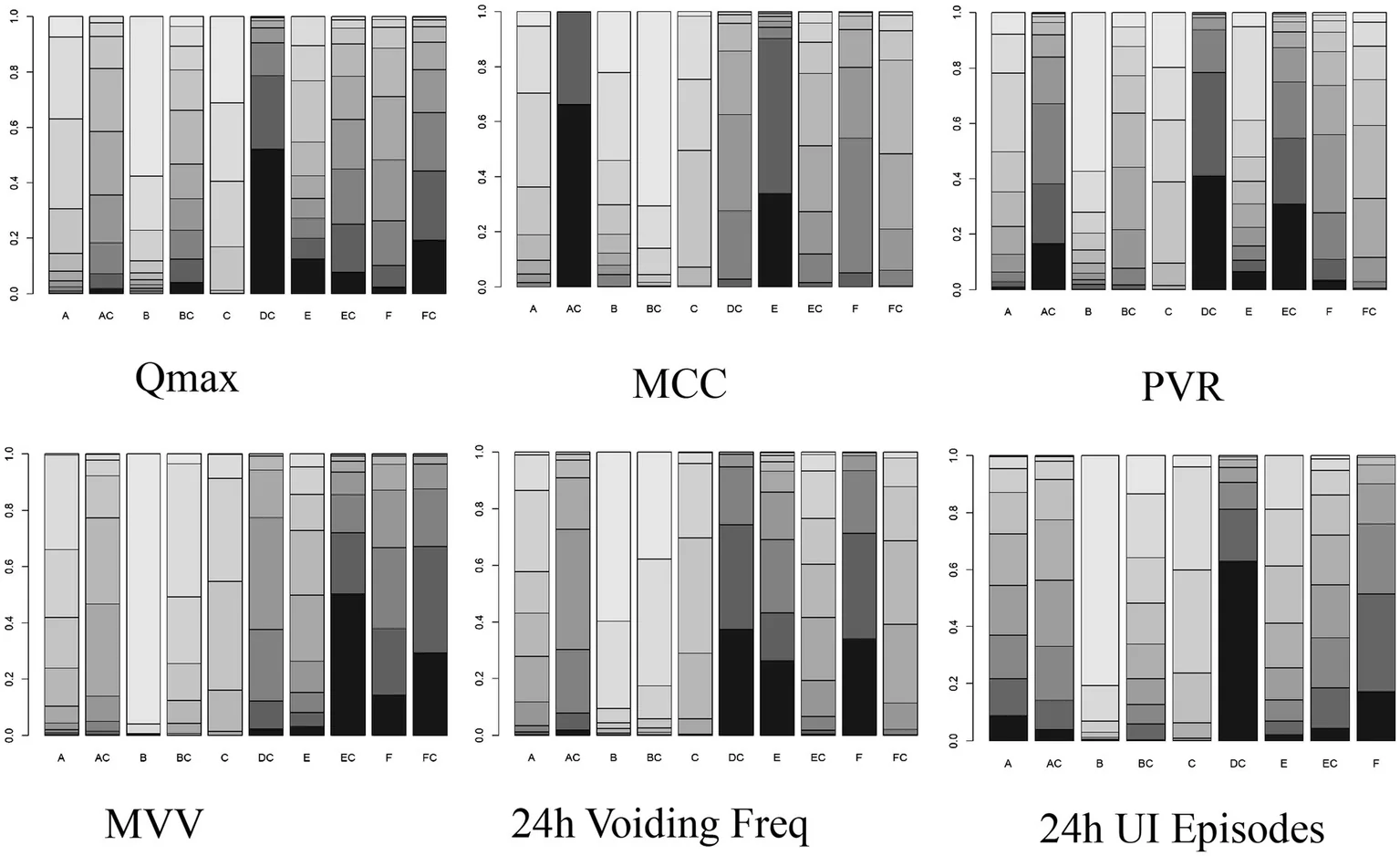
Ranking plot of the effects of different interventions on each outcome indicator. The letters on the horizontal axis represent different interventions, and the vertical axis represents the cumulative probability of each intervention being ranked as the best for the corresponding outcome indicator. Qmax, Maximum urinary flow rate; MCC, Maximum cystometric capacity; PVR, Post-void residual volume; MVV, Mean voided volume; 24 h Voiding Freq, 24-h voiding frequency; 24 h UI Episodes, 24-h urinary incontinence episodes; A, Sacral neuromodulation; AC, Sacral neuromodulation combined with conventional therapy; B, Placebo; BC, Placebo combined with conventional therapy; C, Conventional therapy; DC, Neuromuscular electrical stimulation combined with conventional therapy; E, Tibial nerve stimulation; EC, Tibial nerve stimulation combined with conventional therapy; F, Transcutaneous electrical nerve stimulation; FC, Transcutaneous electrical nerve stimulation combined with conventional therapy.

**Table 2:** Ranking table of cumulative probabilities.

Outcome indicators	A	AC	B	BC	C	DC	E	EC	F	FC
Qmax	0.2414264	0.5479111	0.1066944	0.5032847	0.1418361	0.9057833	0.5084375	0.6705097	0.6018458	0.7722708
MCC	0.26220556	0.96212639	0.21929028	0.05569861	0.25687083	0.63666389	0.90227778	0.50460833	0.70055000	0.49970833
PVR	0.3359486	0.7682708	0.1408194	0.4453556	0.2126347	0.9004861	0.3646000	0.8170264	0.6073319	0.4075264
MVV	0.276897222	0.483001389	0.00579444	0.20909722	0.292456944	0.690930556	0.510359722	0.886373611	0.780005556	0.865083333
24 h Voiding Freq	0.36750694	0.66641944	0.06395139	0.09934444	0.33403194	0.89506806	0.79202778	0.44338194	0.88583194	0.45243611
24 h UI Episodes	0.59594375	0.59267188	0.03877031	0.34196719	0.23366250	0.91051563	0.41582969	0.58232656	0.78831250	-

“–” Indicates that the intervention was excluded from this outcome measure. Each value represents the cumulative probability of the corresponding intervention for a specific outcome measure. Qmax, Maximum urinary flow rate; MCC, Maximum cystometric capacity; PVR, Post-void residual volume; MVV, Mean voided volume; 24 h Voiding Freq, 24-h voiding frequency; 24 h UI Episodes, 24-h urinary incontinence episodes; A, Sacral neuromodulation; AC, Sacral neuromodulation combined with conventional therapy; B, Placebo; BC, Placebo combined with conventional therapy; C, Conventional therapy; DC, Neuromuscular electrical stimulation combined with conventional therapy; E, Tibial nerve stimulation; EC, Tibial nerve stimulation combined with conventional therapy; F, Transcutaneous electrical nerve stimulation; FC, Transcutaneous electrical nerve stimulation combined with conventional therapy.

#### 3.6.2 MCC analysis results

Twenty-four studies reported MCC (12–18, 22, 23, 26–29, 31, 33–37, 39–43). For MCC, the analysis results showed that compared with SNM combined with conventional therapy, SNM (MD = 220 mL; 95% CrI = 110, 320), placebo (MD = 230 mL; 95% CrI = 90, 370), placebo combined with conventional therapy (MD = 280 mL; 95% CrI = 170, 390), conventional therapy (MD = 210 mL; 95% CrI = 160, 260), NMES combined with conventional therapy (MD = 140 mL; 95% CrI = 70, 210), TNS combined with conventional therapy (MD = 160 mL; 95% CrI = 76, 250), TENS (MD = 130 mL; 95% CrI = 63, 190), and TENS combined with conventional therapy (MD = 160 mL; 95% CrI = 96, 230) all performed significantly worse. Compared with conventional therapy, NMES combined with conventional therapy (MD = 65 mL; 95% CrI = 16, 110), TNS (MD = 180 mL; 95% CrI = 42, 310), and TENS (MD = 77 mL; 95% CrI = 36, 120) all performed significantly better. Compared with placebo combined with conventional therapy, SNM combined with conventional therapy (MD = 280 mL; 95% CrI = 170, 390), NMES combined with conventional therapy (MD = 140 mL; 95% CrI = 50, 230), TNS combined with conventional therapy (MD = 120 mL; 95% CrI = 28, 210), TENS (MD = 150 mL; 95% CrI = 47, 260), and TENS combined with conventional therapy (MD = 120 mL; 95% CrI = 9.4, 220) all performed significantly better. Compared with TNS, SNM (MD = 190 mL; 95% CrI = 85, 290), placebo (MD = 200 mL; 95% CrI = 150, 250), and placebo combined with conventional therapy (MD = 250 mL; 95% CrI = 86, 420) all performed significantly worse. No statistically significant differences were observed among other comparisons (see Figure 6). According to the SUCRA results: SNM combined with conventional therapy had the highest probability (96.2%), followed by TNS (90.2%), TENS (70%), NMES combined with conventional therapy (63.6%), TNS combined with conventional therapy (50.4%), TENS combined with conventional therapy (49.9%), SNM (26.2%), conventional therapy (25.6%), placebo (21.9%), and placebo combined with conventional therapy (5.5%) (see Figure 7 and Table 2).

#### 3.6.3 PVR analysis results

Twenty-seven studies reported post-void residual volume (PVR) (12, 13, 15–29, 31, 33, 34, 36–38, 40–43). For PVR, the analysis results showed that compared with conventional therapy, SNM combined with conventional therapy (MD = −36 mL; 95% CrI = −62, −11), NMES combined with conventional therapy (MD = −45 mL; 95% CrI = −61, −30), and TNS combined with conventional therapy (MD = −41 mL; 95% CrI = −72, −8.6) all performed significantly better. NMES combined with conventional therapy was significantly better than placebo combined with conventional therapy (MD = −31 mL; 95% CrI = −58, −5.4) and TENS combined with conventional therapy (MD = −34 mL; 95% CrI = −58, −9.6). No statistically significant differences were observed among other comparisons (see Figure 6). According to the SUCRA results: NMES combined with conventional therapy had the highest probability (90%), followed by TNS combined with conventional therapy (81.7%), SNM combined with conventional therapy (76.8%), TENS (60.7%), placebo combined with conventional therapy (44.5%), TENS combined with conventional therapy (40.7%), TNS (36.4%), SNM (33.5%), conventional therapy (21.2%), and placebo (14%) (see Figure 7 and Table 2).

#### 3.6.4 MVV analysis results

Twenty-eight studies reported MVV (12–26, 28–31, 33, 34, 36–43). For MVV, the analysis results showed that compared with placebo, SNM (MD = 100 mL; 95% CrI = 24, 180), SNM combined with conventional therapy (MD = 140 mL; 95% CrI = 46, 230), conventional therapy (MD = 110 mL; 95% CrI = 29, 190), NMES combined with conventional therapy (MD = 170 mL; 95% CrI = 83, 260), TNS (MD = 140 mL; 95% CrI = 90, 190), TNS combined with conventional therapy (MD = 200 mL; 95% CrI = 100, 310), TENS (MD = 180 mL; 95% CrI = 90, 270), and TENS combined with conventional therapy (MD = 200 mL; 95% CrI = 110, 290) all performed significantly better. Compared with conventional therapy, NMES combined with conventional therapy (MD = 60 mL; 95% CrI = 26, 95), TNS combined with conventional therapy (MD = 95 mL; 95% CrI = 29, 160), TENS (MD = 73 mL; 95% CrI = 28, 120), and TENS combined with conventional therapy (MD = 86 mL; 95% CrI = 45, 130) all performed significantly better. Compared with placebo combined with conventional therapy, NMES combined with conventional therapy (MD = 79 mL; 95% CrI = 23, 140), TNS combined with conventional therapy (MD = 110 mL; 95% CrI = 39, 190), TENS (MD = 92 mL; 95% CrI = 18, 170), and TENS combined with conventional therapy (MD = 110 mL; 95% CrI = 43, 170) all performed significantly better. Compared with SNM, TENS combined with conventional therapy (MD = 94 mL; 95% CrI = 9.9, 180) and TNS combined with conventional therapy (MD = 100 mL; 95% CrI = 2.4, 200) both performed significantly better. No statistically significant differences were observed among other comparisons (see Figure 6).

According to the SUCRA results: TNS combined with conventional therapy had the highest probability (88.6%), followed by TENS combined with conventional therapy (86.5%), TENS (78%), NMES combined with conventional therapy (69%), TNS (51%), SNM combined with conventional therapy (48.3%), conventional therapy (29.2%), SNM (27.6%), placebo combined with conventional therapy (20.9%), and placebo (0.5%) (see Figure 7 and Table 2).

#### 3.6.5 24-h voiding frequency analysis results

Twenty-seven studies reported 24-h voiding frequency (12–17, 19, 20, 23, 24, 26, 28–43). For 24-h voiding frequency, the analysis results showed that compared with placebo, SNM combined with conventional therapy (MD = −4.2; 95% CrI = −7.6, −0.86), NMES combined with conventional therapy (MD = −5.7; 95% CrI = −9.1, −2.4), TNS (MD = −5.1; 95% CrI = −6.8, −3.5), and TENS (MD = −5.6; 95% CrI = −9.0, −2.4) all performed significantly better. Compared with conventional therapy, TENS (MD = −3.2; 95% CrI = −4.6, −1.8) and SNM combined with conventional therapy (MD = −1.7; 95% CrI = −3.3, −0.19) both performed significantly better. Compared with NMES combined with conventional therapy, SNM (MD = −3.2; 95% CrI = −6.3, −0.16), conventional therapy (MD = −3.2; 95% CrI = −4.6, −1.8), TNS combined with conventional therapy (MD = −2.7; 95% CrI = −5.2, −0.35), and TENS combined with conventional therapy (MD = −2.7; 95% CrI = −4.6, −0.81) all performed significantly worse. Compared with placebo combined with conventional therapy, SNM combined with conventional therapy (MD = −3.7; 95% CrI = −6.6, −0.80), NMES combined with conventional therapy (MD = −5.2; 95% CrI = −7.7, −2.7), TNS (MD = −4.6; 95% CrI = −8.4, −0.77), TENS (MD = −5.1; 95% CrI = −8, −2.3), and TENS combined with conventional therapy (MD = −2.5; 95% CrI = −4.9, −0.032) all performed significantly better. No statistically significant differences were observed among other comparisons (see Figure 6).

According to the SUCRA results: NMES combined with conventional therapy had the highest probability (89.5%), followed by TENS (88.5%), TNS (79.2%), SNM combined with conventional therapy (66.6%), TENS combined with conventional therapy (45.2%), TNS combined with conventional therapy (44.3%), SNM (36.7%), conventional therapy (33.4%), placebo combined with conventional therapy (9.9%), and placebo (6.3%) (see Figure 7 and Table 2).

#### 3.6.6 24-h urinary incontinence episodes analysis results

Twenty studies reported 24-h urinary incontinence episodes (12, 13, 15–17, 24–37, 39). For 24-h urinary incontinence episodes, the analysis results showed that compared with placebo, SNM (MD = −2.3; 95% CrI = −4.2, −0.54), SNM combined with conventional therapy (MD = −2.3; 95% CrI = −4.6, −0.13), NMES combined with conventional therapy (MD = −3.5; 95% CrI = −6.1, −0.98), TNS (MD = −1.8; 95% CrI = −2.7, −0.84), and TENS (MD = −2.9; 95% CrI = −5.0, −0.83) all performed significantly better. TENS performed significantly better than conventional therapy (MD = −1.7; 95% CrI = −2.6, −0.72). Compared with NMES combined with conventional therapy, placebo combined with conventional therapy (MD = −2.0; 95% CrI = −3.9, −0.45) and conventional therapy (MD = −2.3; 95% CrI = −4.1, −0.53) both performed significantly worse. No statistically significant differences were observed among other comparisons (see Figure 6).

According to the SUCRA results: NMES combined with conventional therapy had the highest probability (91%), followed by TENS (78.8%), SNM (59.5%), SNM combined with conventional therapy (59.2%), TNS combined with conventional therapy (58.2%), TNS (41.5%), placebo combined with conventional therapy (34.1%), conventional therapy (23.3%), and placebo (3.8%) (see Figure 7 and Table 2).

### 3.7 Publication bias assessment

To assess the extent to which the above comparison results were affected by small-study effects, we performed small-study effect tests for all outcome measures. As shown in Figure 8, the *p* values of the Egger test for each outcome were all ≥ 0.05, with no statistical significance. This indicates that the likelihood of publication bias in these analyses was relatively low.

**Figure 8:**
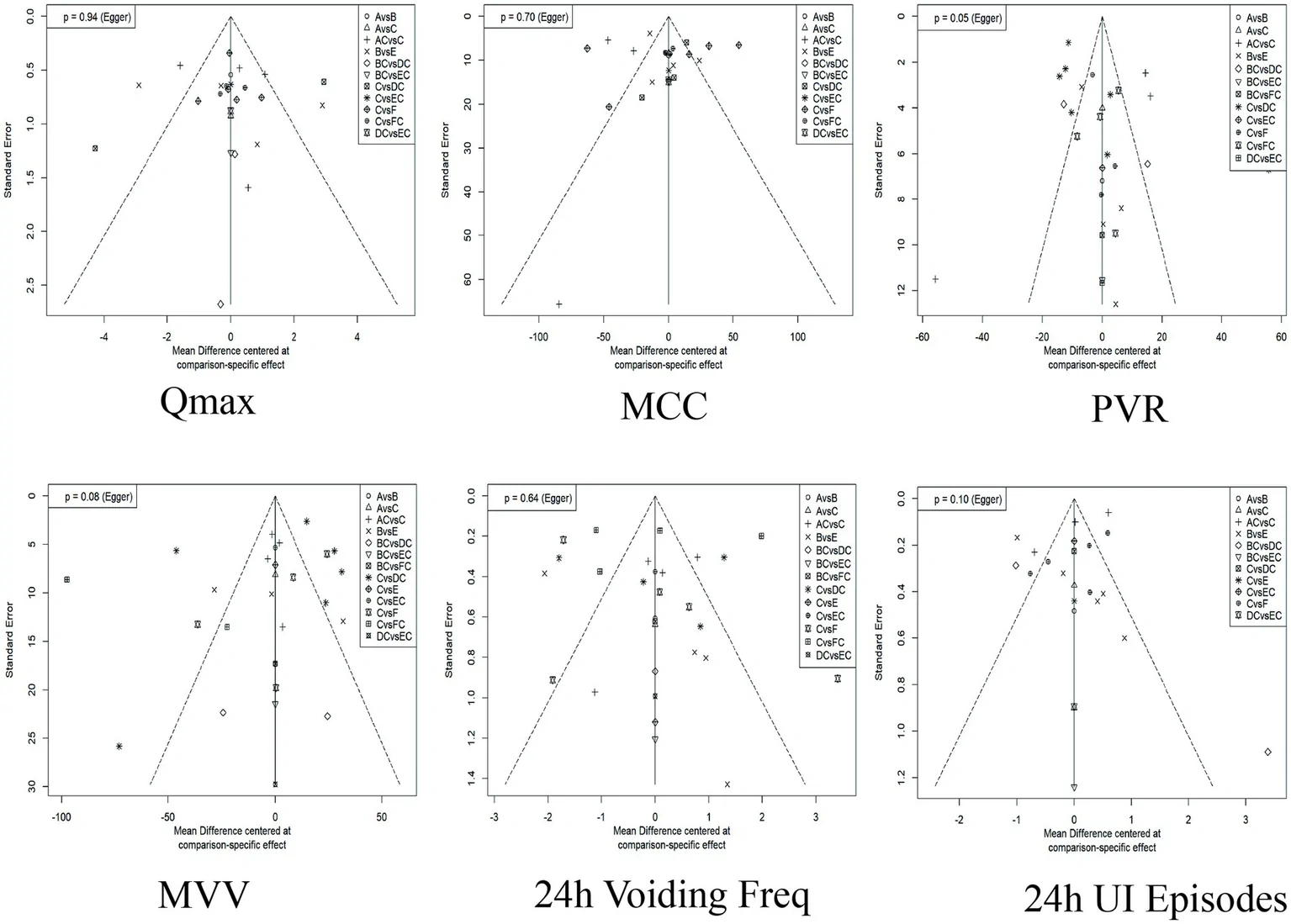
Adjusted funnel plot for each outcome indicator. The horizontal axis represents the mean difference based on specific comparisons, reflecting the effect size differences between interventions. The vertical axis represents the standard error, indicating the precision of the effect estimate; a smaller standard error corresponds to a more precise estimate. Qmax, Maximum urinary flow rate; MCC, Maximum cystometric capacity; PVR, Post-void residual volume; MVV, Mean voided volume; 24 h Voiding Freq, 24-h voiding frequency; 24 h UI Episodes, 24-h urinary incontinence episodes; A, Sacral neuromodulation; AC, Sacral neuromodulation combined with conventional therapy; B, Placebo; BC, Placebo combined with conventional therapy; C, Conventional therapy; DC, Neuromuscular electrical stimulation combined with conventional therapy; E, Tibial nerve stimulation; EC, Tibial nerve stimulation combined with conventional therapy; F, Transcutaneous electrical nerve stimulation; FC, Transcutaneous electrical nerve stimulation combined with conventional therapy.

## 4 Discussion

This study systematically compared the efficacy of multiple neuromodulation strategies on voiding-related parameters in patients with NLUTD based on a Bayesian network meta-analysis. The results showed that neuromuscular electrical stimulation (NMES) combined with conventional therapy had the highest surface under the cumulative ranking curve (SUCRA) values for maximum flow rate (Qmax), post-void residual volume (PVR), 24-h voiding frequency, and 24-h incontinence episodes (90.5, 90.0, 89.5, and 91.0%, respectively); sacral neuromodulation (SNM) combined with conventional therapy had the highest SUCRA for maximum cystometric capacity (MCC) (96.2%); and tibial nerve stimulation (TNS) combined with conventional therapy had the highest SUCRA for mean voided volume (MVV) (88.6%). It should be emphasized that these rankings only reflect relative probability rankings under the current evidence network, and their clinical significance should be interpreted cautiously in conjunction with evidence quality and heterogeneity.

Qmax and PVR are core urodynamic parameters for evaluating bladder emptying efficiency during the voiding phase, and their improvement directly reflects the recovery of voiding function; 24-h voiding frequency and incontinence episodes reflect the impact of storage symptoms on daily life from a patient-reported perspective. NMES combined with conventional therapy ranked highest for all four indicators, suggesting that it may simultaneously improve voiding efficiency and storage function. Mechanistically, NMES can directly stimulate the pelvic floor muscles and surrounding nerves via vaginal/anal probes or surface electrodes, enhancing pelvic floor support structures and external urethral sphincter tone, thereby improving storage symptoms (44); its afferent signals can also be transmitted upward via spinal reflex arcs, inhibit abnormal bladder reflex activity, modulate detrusor contraction, and promote bladder emptying during the voiding phase (45, 46). This dual regulatory mechanism is consistent with the multi-indicator improvement observed in this study.

It is worth emphasizing that the aforementioned benefits are not the result of NMES alone; its combination with conventional therapy may produce synergistic enhancement effects. Conventional therapy (such as intermittent catheterization, pelvic floor muscle training, or pharmacotherapy) focuses on enhancing patients’ conscious control of lower urinary tract function and central integration capacity, whereas NMES enriches peripheral afferent signals for central neuroplastic changes by enhancing peripheral sensory and motor input. Functional near-infrared spectroscopy studies have shown that when NMES is applied simultaneously with active rehabilitation strategies based on mirror neuron theory, it can significantly activate multiple cortical regions including Brodmann areas 6, 40, 44, 45, and 46, with activation extent and intensity superior to single interventions (47), suggesting that combined “peripheral-central” regulation may achieve synergistic improvement in lower urinary tract function. In addition, a meta-analysis by Ali et al. (48) showed that NMES significantly improved urge urinary incontinence symptoms in patients with stroke-related neurogenic bladder (SMD = −2.637; 95% CI: −3.804, −1.474); a consensus report by Çetinel et al. (49) also recommended that when antimuscarinic drug therapy fails or causes severe side effects, pelvic floor rehabilitation combined with NMES should be used to improve treatment success rates. The above evidence is consistent with the NMES efficacy signal observed in this study.

MCC is a core urodynamic parameter for evaluating bladder storage function, reflecting the bladder’s capacity to accommodate urine before reaching maximum tolerated desire to void. Patients with NLUTD often have reduced MCC due to detrusor overactivity or decreased bladder wall compliance, and may develop upper urinary tract dysfunction due to chronically elevated intravesical pressure (50). This study showed that SNM combined with conventional therapy had a SUCRA of 96.2% for MCC, suggesting that it may have a potential advantage in increasing bladder capacity. SNM increases functional bladder capacity by stimulating sacral somatic afferent fibers, inhibiting bladder parasympathetic preganglionic neurons and pelvic nerve efferent activity, activating spinal interneurons that coordinate bladder-sphincter function, and inhibiting C-fiber-mediated sensory pathways, without directly stimulating detrusor or urethral sphincter motor neurons (51). Han et al. (52) reported a patient with NLUTD caused by organophosphate-induced delayed neuropathy, whose bladder capacity increased from 150 mL to 320 mL after SNM treatment; Chen Guoqing et al. (53) reported 33 cases of neurogenic bladder and bowel dysfunction associated with spina bifida treated with SNM, with an overall success rate of 69.69% at 14–28 days after treatment, increased maximum cystometric capacity and bladder compliance, and decreased maximum detrusor pressure during filling. These studies support the potential role of SNM in improving MCC. However, it should be emphasized that only six SNM-related RCTs were included in this study, and direct comparative evidence between SNM and other interventions is limited; therefore, this result should be regarded as an exploratory preliminary finding and cannot yet confirm that SNM combined therapy is superior to other neuromodulation techniques.

MVV is derived from voiding diaries and reflects patients’ actual voiding patterns in daily life, better representing true bladder function than laboratory urodynamic parameters. This study showed that TNS combined with conventional therapy had the highest SUCRA for MVV (88.6%), suggesting that it may be a preferred strategy for improving daily voiding patterns in patients with NLUTD. TNS stimulates the posterior tibial nerve, transmitting sensory afferent signals via the sciatic nerve to the sacral spinal cord, activating somato-visceral reflex inhibitory pathways at the spinal level, reducing detrusor hyperexcitability, prolonging the voiding interval, and thereby increasing the volume voided per micturition (54, 55). Animal experiments have shown that TNS can activate the expression of transient receptor potential vanilloid type 1 (TRPV1) channel protein (56). TRPV1, as a mechanogated channel, senses mechanical stretch in bladder sensory neurons and transmits filling signals, and may participate in voiding regulation (57). A meta-analysis by Fang Zheng (58) including 314 patients with spinal cord injury, multiple sclerosis, Parkinson’s disease, or stroke showed that TNS significantly improved bladder capacity (*p* = 0.0005), post-void residual volume (*p* < 0.00001), and mean voided volume (*p* = 0.003), which is generally consistent with the results of this study. Furthermore, Ergin et al. (59) compared once-weekly and twice-weekly TNS for overactive bladder and found that the overall efficacy at 12 weeks was similar between the two groups, but the twice-weekly group showed faster early symptom improvement, suggesting that stimulation frequency may mainly affect the speed of onset rather than final efficacy. The TNS-related studies included in this study differed in treatment frequency, duration of each session, and total treatment course. Such within-protocol heterogeneity may have a certain impact on the pooled effect estimates and ranking results, especially in the assessment of early efficacy.

TENS, by contrast, typically uses surface electrodes to stimulate peripheral nerves or corresponding dermatomes, with a relatively broad range of action, and may influence bladder reflexes through both segmental spinal inhibition and supraspinal central regulation (60). TNS, TENS, and NMES are all forms of electrical stimulation; however, because they differ in stimulation targets and neuromodulatory pathways, their improvements in storage symptoms and voiding parameters may have different emphases. Furthermore, because they differ in application site, stimulation parameters, and treatment frequency, although SUCRA rankings may suggest potential advantages of each electrical stimulation modality for different outcomes, direct comparative evidence among the different techniques is relatively limited, and the ranking results should be interpreted cautiously in the context of heterogeneity in intervention implementation.

It should be noted that the randomized controlled trials included in this study had some heterogeneity in patient clinical characteristics, which may have influenced the pooled effect estimates and ranking results. In terms of neurological etiology, the included patients had neurogenic lower urinary tract dysfunction caused by various etiologies, including spinal cord injury, multiple sclerosis, Parkinson’s disease, stroke, and spina bifida. The pathophysiological mechanisms of lower urinary tract dysfunction caused by different etiologies are not entirely identical: spinal cord injury often manifests as detrusor overactivity with detrusor-sphincter dyssynergia (61), whereas central neurodegenerative diseases such as multiple sclerosis and Parkinson’s disease are predominantly characterized by detrusor overactivity (62); the direction and degree of impact on storage and voiding function may differ among etiologies. Age and disease severity, such as disease duration, baseline symptom scores, and degree of urodynamic impairment, also affect neuroplasticity and pelvic floor muscle functional reserve, thereby altering patients’ response to neuromodulation therapy. Baseline urodynamic characteristics, including maximum cystometric capacity, bladder compliance, detrusor pressure, and the presence of detrusor overactivity, directly determine the baseline risk of the populations included in each study; some studies may have focused primarily on storage symptoms, while others may have focused on voiding dysfunction. Such baseline differences directly affect the magnitude of improvement in Qmax, PVR, MCC, and other parameters, as well as comparability among studies. In terms of treatment duration, studies differed in intervention course and follow-up length; shorter courses may be insufficient to fully manifest neural remodeling effects, whereas longer courses may amplify between-group differences. In addition, the conventional therapy node internally includes multiple interventions such as pharmacotherapy, pelvic floor muscle training, and intermittent catheterization, whose mechanisms of action and effect sizes are not entirely consistent, potentially introducing within-category heterogeneity. Although these interventions were pooled for analysis due to limited network stability and number of studies, their internal differences may still affect the reliability of some indirect comparisons. At the level of stimulation parameters, the included studies varied considerably in electrical stimulation frequency (10–75 Hz), pulse width (70–450 μs), and session duration (20–40 min). Stimulation frequency, pulse width, and treatment duration are key parameters affecting the effects of nerve electrical stimulation; different parameter combinations may alter activation thresholds, recruitment patterns, and degree of neuroplastic changes in nerve fibers, thereby influencing the magnitude of improvement in lower urinary tract function. Therefore, the observed efficacy differences among different interventions in this study may reflect both the mechanistic characteristics of the different electrical stimulation modalities themselves and, in part, differences in stimulation parameter selection across studies.

The above clinical and methodological heterogeneity reduces comparability among studies and weakens the strength of evidence from indirect comparisons on the one hand, and may increase uncertainty in ranking probabilities on the other. Nevertheless, NMES combined with conventional therapy showed relatively consistent advantages across multiple voiding-related parameters, suggesting that its efficacy signal may have some cross-etiology stability; however, due to the lack of individual patient data and sufficient subgroup information, stratified analyses for the above confounding factors could not be performed.

Although this study yielded relevant conclusions, it still has the following limitations:

1. The included studies had clinical heterogeneity in neurological etiology, age, disease severity, baseline urodynamic characteristics, and treatment duration, which may reduce comparability among studies.
2. The route of NMES administration differed in some studies, such as vaginal probes and anal probes. Although this study uniformly classified them as NMES, such within-intervention heterogeneity may affect the interpretation of results. Because the original studies did not report sufficient details, subgroup analyses by route of administration could not be performed.
3. The conventional therapy group included multiple intervention modalities, such as pharmacotherapy, pelvic floor muscle training, and intermittent catheterization. Pooled analysis may introduce within-category heterogeneity; although Table 1 provides specific regimens, this heterogeneity may still affect the reliability of some comparisons.
4. The electrical stimulation parameters varied considerably among the included studies, with a frequency range of 10–75 Hz, pulse width of 70–450 μs, and session duration of 20–40 min. This parameter heterogeneity may introduce within-node heterogeneity and affect the reliability of efficacy comparisons among different electrical stimulation modalities.
5. Only six SNM-related studies met the inclusion criteria, resulting in sparse direct comparative evidence between SNM and other interventions and limited stability and reliability of efficacy estimates. Therefore, SNM-related results should be regarded only as preliminary exploratory evidence and should not be interpreted as definitive efficacy superiority. In addition, differences in application site, stimulation target, and treatment protocol among NMES, TNS, and TENS may also affect the effect estimates of direct and indirect comparisons.
6. A relatively high proportion of the included randomized controlled trials were from China, which may introduce publication bias favoring positive results. Although the Egger test did not show significant small-study effects, the test may have insufficient power due to the limited number of included studies and the presence of clinical heterogeneity; therefore, the impact of publication bias on pooled effect estimates cannot be completely excluded. In addition, the number of studies per comparison was small, with indirect evidence and risk of bias, and the rankings may be unstable. Therefore, the study conclusions require further validation by large multicenter, large-sample, double-blind randomized controlled trials.

## 5 Conclusion

Based on the exploratory ranking results of the surface under the cumulative ranking curve (SUCRA), NMES combined with conventional therapy ranked highest for maximum flow rate, post-void residual volume, 24-h voiding frequency, and 24-h urinary incontinence episodes; SNM combined with conventional therapy ranked highest for maximum cystometric capacity; and TNS combined with conventional therapy ranked highest for mean voided volume. However, these rankings only represent relative probability rankings under the current evidence network and cannot establish definitive superiority of the aforementioned combination regimens over other interventions. Because some outcomes had a relatively small number of included studies and the analyses relied heavily on indirect comparative evidence, the stability of efficacy rankings is insufficient; therefore, the above results should be regarded as preliminary exploratory findings and should be interpreted with great caution.

## Data Availability

The original contributions presented in the study are included in the article/Supplementary material, further inquiries can be directed to the corresponding authors.
