# Supplementary material for: Efficacy differences between SNM and various peripheral nerve electrical stimulation modalities for NLUTD: a Bayesian network meta-analysis
Source: Front Neurol. 2026 Sep 21;17:1958819. doi: 10.3389/fneur.2026.1958819 (PMC13634872; doi:10.3389/fneur.2026.1958819)
Supplement: Supplementary file 1 [file Supplementary_file_1.DOCX]

Supplementary Figure 1 Risk of bias summary

Supplementary Figure 2 Trace plot and density plot for each result indicator

Note:
Trace Plots: The horizontal axis represents the number of iterations, and the vertical axis represents the range of parameter values. The multi-colored curves represent the paths of independent Markov chains, reflecting parameter space exploration and convergence.
Density Plots: The horizontal axis represents possible parameter values. The vertical axis represents probability density. The total area under the curve integrates to 1. A taller and narrower peak indicates a higher likelihood of parameter values in that region.
Qmax: Maximum urinary flow rate; MCC: Maximum cystometric capacity; PVR: Post-void residual volume; MVV: Mean voided volume; 24h Voiding Freq: 24-hour voiding frequency; 24h UI Episodes: 24-hour urinary incontinence episodes; A: Sacral neuromodulation; AC: Sacral neuromodulation combined with conventional therapy; B: Placebo; BC: Placebo combined with conventional therapy; C: Conventional therapy; DC: Neuromuscular electrical stimulation combined with conventional therapy; E: Tibial nerve stimulation; EC: Tibial nerve stimulation combined with conventional therapy; F: Transcutaneous electrical nerve stimulation; FC: Transcutaneous electrical nerve stimulation combined with conventional therapy.

Supplementary Table 1 Literature Search Strategy Table Taking PubMed as an Example

| Databases: | PubMed | |
| --- | --- | --- |
| Search # | Search Query | Search Fields |
| #1 | ("Neurogenic Lower Urinary Tract Dysfunction"[tiab] OR "Neurogenic Bladder"[Mesh] OR "Neurogenic Voiding Dysfunction"[tiab] OR "Neurogenic Urinary Tract Dysfunction"[tiab]) | All fields |
| #2 | ("Sacral Neuromodulation"[Mesh] OR "SNM"[tiab]) | All fields |
| #3 | ("Neuromuscular Electrical Stimulation"[Mesh] OR "NMES"[tiab]) | All fields |
| #4 | ("Tibial Nerve Stimulation"[tiab] OR "Percutaneous Tibial Nerve Stimulation"[tiab] OR "TNS"[tiab]) | All fields |
| #5 | ("Transcutaneous Electrical Nerve Stimulation"[Mesh] OR "TENS"[tiab]) | All fields |
| #6 | #2 OR #3 OR #4 OR #5 | All fields |
| #7 | ("Randomized Controlled Trial"[pt] OR "Controlled Clinical Trial"[pt] OR "Randomized"[tiab] OR "Randomly"[tiab] OR "Placebo"[tiab] OR "Clinical Trial"[pt]) NOT ("Animals"[Mesh] NOT "Humans"[Mesh]) | All fields |
| #8 | #1 AND #6 AND #7 | All fields |

Supplementary Table 2 Summary of Implementation Characteristics of Neuromuscular Electrical Stimulation (NMES) Interventions in Included Studies

| Author (year) | Major Intervention Category | Implementation Approaches | Electrode placement method | target muscle group |
| --- | --- | --- | --- | --- |
| Lúcio 2016 | NMES | Endocavity (vaginal probe) | Periform vaginal probe is inserted into the vaginal fornix, with the intracavitary electrode ring attached to the pelvic floor mucosa | Pelvic floor muscles (levator ani muscle, puborectalis muscle) |
| McClurg 2008 | NMES | Intracavitary (vaginal/anal probe) | Periform Vaginal Probe (Female) / Anuform Anal Probe (Suitable for Both Males and Females) | Pelvic floor muscles (levator ani / external anal sphincter) |
| DM Xu 2015 | NMES | Intracavity (anal probe) | Intracavitary electrode rod is inserted into the anus to fit the pelvic floor mucosa | external anal sphincter |
| LT Chen 2016 | NMES | Intracavitary (vaginal/anal probe) | Intracavitary electrode rod is inserted into the anus or vagina to fit against the pelvic floor mucosa | Pelvic floor muscles (levator ani / external anal sphincter |
| SX Chen 2014 | NMES | Endocavity (vaginal probe) | Insert the intracavitary electrode probe into the anus or vagina to fit the pelvic floor mucosa. | Pelvic floor muscles (levator ani / external anal sphincter |
| HQ Zhou 2020 | NMES | Endocavity (vaginal probe) | Insert the electrode rod 5‑7 cm into the vagina (for females) or anus (for both males and females) | Pelvic floor muscles (levator ani / external anal sphincter |
| SG Pan 2016 | NMES | Intracavitary (vaginal/anal probe) | Insert the electrode rod 5‑7 cm into the vagina (for females) or anus (for both males and females) | Pelvic floor muscles (levator ani / external anal sphincter |
| XL An 2014 | NMES | Intracavity (anal probe) | Intracavitary electrode rod is inserted into the anus to fit the pelvic floor mucosa | external anal sphincter |
| ZL Ma 2021 | NMES | Intracavitary (vaginal/anal probe) | Insert the electrode rod 5‑7 cm into the vagina (for females) or anus (for both males and females) | Pelvic floor muscles (levator ani / external anal sphincter |

Supplementary Table 3 GRADE Assessment Results Table, Qmax: maximum urinary flow rate; MCC: maximum cystometric capacity; PVR: post-void residual urine volume; MVV: mean voided volume per micturition; 24h Voiding Freq: 24-hour voiding frequency; 24h UI Episodes: 24-hour urinary incontinence episodes.

| Outcome | Studies, n | Study design | Risk of bias | Inconsistency | Indirectness | Imprecision | publication bias | Certainty |
| --- | --- | --- | --- | --- | --- | --- | --- | --- |
| Qmax | 23 | RCT | serious | Not serious | Not serious | Not serious | Not serious | Moderate |
| MCC | 24 | RCT | Not serious | Not serious | Not serious | serious | Not serious | Moderate |
| PVR | 27 | RCT | Not serious | Not serious | Not serious | serious | Not serious | Moderate |
| MVV | 28 | RCT | serious | Not serious | Not serious | Not serious | Not serious | Moderate |
| 24h Voiding Freq | 27 | RCT | serious | Not serious | Not serious | Not serious | Not serious | Moderate |
| 24h UI Episodes | 20 | RCT | serious | Not serious | Not serious | Not serious | Not serious | Moderate |

Supplementary Table 4 Goodness-of-fit and heterogeneity assessment results of consistency models under different outcome indicators

Note: DIC = Deviance Information Criterion; Dbar = posterior mean deviance; pD = effective number of parameters; Qmax = maximum urinary flow rate; MCC = maximum cystometric capacity; PVR = post-void residual urine volume; MVV = mean voided volume per micturition; 24h Voiding Freq = 24-hour voiding frequency; 24h UI Episodes = 24-hour urinary incontinence episodes.

| Outcome indicators | Model Type | Dbar | pD | DIC | I^2^ | Model selection |
| --- | --- | --- | --- | --- | --- | --- |
| Qmax | Random Effects Model | 47.12713 | 44.36459 | 91.49172 | 2% | Random Effects Model |
|  | Fixed Effects Model | 114.53018 | 32.11207 | 146.64225 | 60% |  |
| MCC | Random Effects Model | 49.05489 | 47.60759 | 96.66248 | 2% | Random Effects Model |
|  | Fixed Effects Model | 438.20093 | 33.81482 | 472.01575 | 89% |  |
| PVR | Random Effects Model | 55.96089 | 53.47569 | 109.43658 | 4% | Random Effects Model |
|  | Fixed Effects Model | 226.30322 | 36.07277 | 262.37599 | 76% |  |
| MVV | Random Effects Model | 57.05681 | 55.54126 | 112.59807 | 2% | Random Effects Model |
|  | Fixed Effects Model | 544.8726 | 37.0777 | 581.9503 | 90% |  |
| 24h Voiding Freq | Random Effects Model | 55.29771 | 52.33446 | 107.63217 | 2% | Random Effects Model |
|  | Fixed Effects Model | 320.94840 | 36.15656 | 357.10496 | 83% |  |
| 24h UI Episodes | Random Effects Model | 43.09654 | 39.40328 | 82.49982 | 7% | Random Effects Model |
|  | Fixed Effects Model | 154.95499 | 28.33516 | 183.29015 | 74% |  |
